# Joint effects of ethnic enclave residence and ambient volatile organic compounds exposure on risk of gestational diabetes mellitus among Asian/Pacific Islander women in the United States

**DOI:** 10.1186/s12940-021-00738-7

**Published:** 2021-05-08

**Authors:** Andrew D. Williams, Sandie Ha, Edmond Shenassa, Lynne C. Messer, Jenna Kanner, Pauline Mendola

**Affiliations:** 1 Public Health program, Department of Population Health, School of Medicine & Health Sciences, University of North Dakota, Room E162, 1301 North Columbia Road Stop 9037, Grand Forks, ND 58202-9037 USA; 2 School of Social Sciences, Humanities and Arts, Health Science Research Institute, University of California, 5200 N. Lake Road, Merced, CA USA; 3 Maternal and Child Health Program, Department of Family Science, University of Maryland College Park, 4200 Valley Drive, College Park, MD USA; 4 OHSU-PSU School of Public Health, Portland State University, 506 SW Mill Street 470H, Portland, OR USA; 5 Epidemiology Branch, Division of Intramural Population Health Research, Eunice Kennedy Shriver National Institute of Child Health and Human Development, 6710B Rockledge Drive, MSC, Bethesda, MD 7004 USA

**Keywords:** Air pollution, Asian/Pacific Islanders, Gestational Diabetes Mellitus, Pregnancy, Volatile Organic Compounds, Ethnic enclave, Stress, Joint exposure

## Abstract

**Background:**

Asian/Pacific Islander (API) communities in the United States often reside in metropolitan areas with distinct social and environmental attributes. Residence in an ethnic enclave, a socially distinct area, is associated with lower gestational diabetes mellitus (GDM) risk, yet exposure to high levels of air pollution, including volatile organic compounds (VOCS), is associated with increased GDM risk. We examined the joint effects of ethnic enclaves and VOCs to better understand GDM risk among API women, the group with the highest prevalence of GDM.

**Methods:**

We examined 9069 API births in the Consortium on Safe Labor (19 hospitals, 2002–2008). API ethnic enclaves were defined as areas ≥66th percentile for percent API residents, dissimilarity (geographic dispersal of API and White residents), and isolation (degree that API individuals interact with another API individual). High levels of 14 volatile organic compounds (VOC) were defined as ≥75th percentile. Four joint categories were created for each VOC: Low VOC/Enclave (reference group), Low VOC/No Enclave, High VOC/Enclave, High VOC/No Enclave. GDM was reported in medical records. Hierarchical logistic regression estimated odds ratios (OR) and 95% confidence intervals (95%CI) between joint exposures and GDM, adjusted for maternal factors and area-level poverty. Risk was estimated for 3-months preconception and first trimester exposures.

**Results:**

Enclave residence was associated with lower GDM risk regardless of VOC exposure. Preconception benzene exposure was associated with increased risk when women resided outside enclaves [High VOC/No Enclave (OR:3.45, 95%CI:1.77,6.72)], and the effect was somewhat mitigated within enclaves, [High VOC/Enclave (OR:2.07, 95%:1.09,3.94)]. Risks were similar for 12 of 14 VOCs during preconception and 10 of 14 during the first trimester.

**Conclusions:**

API residence in non-enclave areas is associated with higher GDM risk, regardless of VOC level. Ethnic enclave residence may mitigate effects of VOC exposure, perhaps due to lower stress levels. The potential benefit of ethnic enclaves warrants further study.

**Supplementary Information:**

The online version contains supplementary material available at 10.1186/s12940-021-00738-7.

In the United States (U.S.), the Asian/Pacific Islander (API) population increased 72% between 2000 and 2015, more than any other racial/ethnic group [1]. Approximately 95% of the U.S. API population is concentrated in metropolitan areas [2], contributing to distinct social and environmental attributes of these areas [3–8]. Despite the concentration of API communities in metropolitan areas, these populations are underrepresented in the environmental health literature, in part due to the ‘model minority’ myth, which due to high average socioeconomic status, suggests API populations have better health outcomes compared to other racial/ethnic groups [6, 9, 10]. The purpose of this study is to build on previous investigations of contextual health determinants [11, 12] among pregnant API women to provide additional data to better understand the health implications of joint social and environmental exposures among U.S. API communities.

Ethnic enclaves are socially and geographically distinct areas with relatively high concentrations of residents of a similar racial/ethnic ancestry within a metropolitan area [3–5]. Evidence suggests that residence in an ethnic enclave may contribute to better health outcomes among members of the prominent group in that area. Compared to API residents of non-enclave areas, API residents of ethnic enclaves live longer [13] and have lower cancer risk [14]. Evidence among pregnant API women is limited, yet initial findings suggest API women residing in ethnic enclaves seek prenatal care earlier [15] and smoke and use alcohol at lower rates [11, 15, 16]. However, the potentially healthy effect of residence in an ethnic enclave may not be uniform, and may differ by ancestry of the API population. For example, among API populations in the United Kingdom-based Millennium Cohort, ethnic enclave residence increased risk of low birth weight birth among Bangladeshi and Indian mothers, yet was associated with reduced risk of low birth weight among Pakistani mothers [17]. The potential effect of ethnic enclaves among API populations is unclear regarding preterm birth and gestational diabetes mellitus [11, 17, 18]. The potentially healthy effect observed among residents of ethnic enclaves, compared to residents in non-enclave areas is hypothesized to be due to low exposure to discrimination [19, 20] and stress [19], which are among the key determinants of health [21–23]. The reduced exposure to discrimination and stress among ethnic enclave residents is likely due to residents’ high levels of political representation and civic participation, as well as greater access to culturally-relevant goods and services that maintains the resident population’s connection to their cultural identity [3–5, 15, 24].

Compared to white communities, communities of color are overburdened with air pollution exposure in the U.S. [6–8, 25] On average, API communities are exposed to similarly high levels of air pollution in comparison to Black and Hispanic communities [6–8, 25]. Additionally, pregnant API women are nearly three times as likely to live in areas with high levels of air pollution compared to pregnant white women [25]. Exposure to high levels of various types of air pollution is associated with systemic inflammation and oxidative stress, which may contribute to poor health outcomes [26–31]. Evidence from animal studies suggest VOCs also induce systemic inflammation and oxidative stress. For instance, among rats, increasing exposure to benzene, a volatile organic compound (VOC), has a linear association with oxidative stress, pancreatic β-cell dysfunction, and greater insulin resistance [32]. High levels of oxidative stress have been linked with pancreatic β-cell dysfunction and insulin resistance among humans [30, 31]. Furthermore, API populations have a higher prevalence of genetic variations associated with pancreatic β-cell dysfunction and insulin resistance than other racial/ethnic groups [33, 34]. While the air pollution-oxidative stress pathway is not specific to API populations, the observed genetic variations may make API populations more susceptible to high levels of air pollution exposure. Thus, the potential interaction between air pollution exposure and social context merits further attention given the potential genetic susceptibility for adverse metabolic outcomes among API populations.

As high exposure to psychosocial stress is associated with immune system dysfunction [35], low exposure to stress among residents of ethnic enclaves suggests more normative immune function. As part of the normative immune response, cells exposed to an insulting agent release pro-inflammatory cytokines and become inflamed in order to isolate damage and protect healthy cells and tissue; as the insult is eliminated, anti-inflammatory cytokines are released and inflammation is contained, thus mitigating development of disease [36]. In contrast, air pollution exposure signals an inflammatory response [27, 32, 37–40], and among those with a compromised immune system, an excessive inflammatory response to air pollution may increase risk for metabolic disease [41, 42]. Considering joint social and environmental exposures among API communities in the U.S. will provide new insights into health outcomes among these understudied populations [43]. To the best of our knowledge, joint exposure to residence in ethnic enclaves and air pollution has not yet been examined in pregnant women. Given evidence suggesting residence in ethnic enclaves may be less stressful residential contexts than other areas [19, 20], residents of ethnic enclaves more normative immune function may better mitigate the negative health consequences associated with exposure to air pollution compared to those residing elsewhere.

Gestational diabetes mellitus (GDM) presents a unique opportunity to examine joint exposures to ethnic enclaves and air pollution among U.S. API women. In the U.S., API women have the highest prevalence of GDM compared to other racial/ethnic groups [12, 44–52]. GDM is associated with an increased risk of maternal, fetal and neonatal complications, including an increased risk of developing type 2 diabetes mellitus among mothers, and increased risk of obesity and diabetes among offspring [53]. Within the Consortium on Safe Labor (CSL), we observed API women had higher prevalence of GDM (9.9%) compared to white (4.5%), Black (4.3%), and Hispanic (6.4%) women [12]. Furthermore, in separate studies among the CSL, API women residing in ethnic enclaves had lower risk of GDM compared to API women residing in non-enclave areas [11] and that exposure to high levels of VOCs early in pregnancy was associated with a greater increase in risk of GDM among API women than among women of other racial/ethnic groups [12]. Thus, we hypothesized that pregnant API women residing in an ethnic enclaves were less susceptible to negative consequences of air pollution than pregnant API women residing elsewhere.

## Methods

### Data and participants

The Consortium on Safe Labor (CSL) was a national, electronic medical record-based retrospective cohort study from 2002 to 2008 which included 19 hospitals (8 university teaching hospitals, 9 community teaching hospitals, 2 community hospitals) in 15 Hospital Referral Regions (HRR), catchment areas for tertiary care hospitals [54]. Hospitals were selected based on availability of electronic medical records, and for representation of the 9 American College of Obstetricians and Gynecologists districts [55]. Data were extracted for deliveries ≥23 weeks gestation and include maternal sociodemographic characteristics; medical, reproductive and prenatal history; labor and delivery, and newborn data. A total of 228,438 deliveries were included in the CSL. We excluded multifetal pregnancies (*n* = 5053; 2.21%), mothers with pre-existing diabetes (*n* = 3309; 1.44%), and those with missing air pollution exposure information (*n* = 10; .004%). Including only API mothers resulted in an analytic sample of 9069 births to 8350 mothers. Institutional Review Boards at all sites approved the CSL, and data are anonymous.

### Outcome variable

GDM was drawn from medical record data or in discharge summaries using ICD-9 code 648.8. During the CSL study period (2002–2008), the American Diabetes Associations recommended screening for GDM between 24 and 28 weeks gestation using the Carpenter and Coustan criteria [56].

### Ethnic enclave exposure

In the CSL, area of residence was estimated using the HRR in which the birth occurred. HRR is the only geographic unit of analysis available in the CSL [57]. HRRs are regional geographies (average miles^2^: 13,065) comparable to Metropolitan Statistical Areas [58], with large enough populations (average population size in thousands: 2026) for observable residential sorting [54, 58].

We aggregated sociodemographic data at the zip code tabulation area (ZCTA) level to provide estimates at the HRR level. As HRR are partially defined by ZCTA, we aggregated ZCTA data to the corresponding HRR using year-specific ZCTA to HRR crosswalk from the Dartmouth Atlas of Health Care [54, 58]. ZCTA data was accessed from the National Historical Geographic Information System for the 2000 decennial census, and the 2007–2011 5-year average of the American Community Survey (ACS) [59]. We linked CSL data with year-specific sociodemographic data: births between 2002 and 2004 were linked with 2000 Census data, and births between 2005 and 2008 were linked with 2007–2011 ACS data [11, 58].

We identified ethnic enclaves at the HRR level [11]. HRRs are centered on urban areas, where the majority of U.S. API populations reside [2], yet the regional coverage of HRRs allows for inclusion of potential ethnic enclaves outside of urban centers [60].

Described in Table 1, the distinct social and geographic attributes of an ethnic enclave are represented by API population density and racial/ethnic segregation, defined using three variables [5, 11]. First, API population density, is measured by the percent of API individuals residing in an HRR. Second, API-White dissimilarity index, is the differential distribution of API and White populations within a geographic area [61, 62]. Lastly, the API isolation index, is the probability that an API individual will interact with another API individual [61, 62]. API population density, API-white dissimilarity index, and API isolation index were calculated separately for Census data and ACS data.

**Table 1:** Area-level measures used to identify ethnic enclaves (also described in Williams et al., 2020) [11]

Measure	Formula	Description
API population density (social attribute)	(*A*_*T*_/*P*_*T*_) ∗ 100	Percentage of API residents within an HRR. Range 0–100; 100 suggests HRR consists of only API residents
Dissimilarity Index (geographic attribute)	$\frac{1}{2}\ \sum \limits_{i=1}^n\ \left|\frac{w_i}{W_T}-\frac{a_i}{A_T}\right|$	Differential distribution of API and White populations within an HRR. Range 0–1; score of 1 suggests absolute geographic separation of API and White populations within HRR.
Isolation Index (geographic attribute)	$\sum \limits_{i=1}^n\ \left(\frac{a_i}{A_T}\right)\ast \left(\frac{a_i}{P_T}\right)$	Probability that API residents of an HRR will interaction with another API individual. Range 0–1; score of 1 suggests an API resident in an HRR will only interact with other API residents.
**Components**	**Description**	
*a*_*i*_	Number of API in the Zip code	
*A*_*T*_	Number of API in the HRR	
*n*	Number of Zip codes	
*P*_*T*_	Total population of the HRR	
*w*_*i*_	Number of white in the Zip code	
*W*_*T*_	Number of white in the HRR	

We used population-based percentiles [4, 5, 11, 18] to identify tertiles (low, medium, high) for API population density, API-white dissimilarity, and API isolation. An HRR was considered an ethnic enclave if it was in the upper third of the distribution for all three variables: API population density, API-white dissimilarity, and API isolation [11].

### Ambient volatile organic compound exposure

The Air Quality and Reproductive Health study estimated VOC exposure in the CSL using a modified version of the Community Multiscale Air Quality Model (version 4.7.1), a 3-dimensional, multipollutant air quality model used to predict ambient pollutant levels using 2005 (version 4) National Emission Inventory (NEI) emissions data and Weather Research Forecasting Model meteorological data. The 2005 NEI v4 was used to generate anthropogenic emissions for 2005–2010. Emissions of year 2002 to 2004 and 2006–2010 were adjusted based on the average annual emissions trends. Modified CMAQ models were evaluated at 4 km and 36 km, and we used 36 km as the HRR resolution was minimally impacted [57]. Exposure was based on predicted hourly ambient pollutant concentrations within HRRs, fused with local air monitoring data to improve accuracy, and weighted to reflect population concentration and non-residential areas (i.e. industrial, large parks, water, mountains), as previously described [57].

As GDM screening is recommended between 24 and 28 weeks gestation [56], we averaged the predicted hourly ambient pollutant concentration across preconception (3 months preconception) and first trimester (through 13 weeks gestation) exposure windows. Ambient concentrations (parts per billion; ppb) were estimated for 14 VOCs: benzene, 1,3-butadiene, ethylbenzene, cyclohexane, methyl-tertiary-butyl ether, *N*-hexane, ethyl-methyl ketone, m-xylene, o-xylene, p-xylene, propene, sesquiterpene, styrene, and toluene for each exposure window. Exposure to ≥75th percentile in ppb was considered high exposure, and all values <75th percentile in ppb were considered low exposure.

### Joint exposure categories

Using the categorical ethnic enclave (yes/no) variable, and the categorical VOC (high/low) variable, we created joint exposure categories: Low VOC/Enclave (reference), Low VOC/No Enclave, High VOC/Enclave, High VOC/No Enclave. The joint exposure variables were created for each of the 14 VOC in both the preconception and first trimester exposure windows.

### Covariates

Individual-level covariates included maternal age, marital status (married, single, other), health insurance (public, private, other), pre-pregnancy body mass index (BMI, < 18.5, 18.5- < 224.9, 25- < 29.9, ≥30), season of conception (winter, spring, summer, fall) and parity (nulliparous or multiparous). As income is not available in the CSL, health insurance [63] and marital status [64] were used as proxies for socioeconomic status. BMI was imputed using multiple imputations (10 iterations) due to a high degree of missingness (42%).

Area-level poverty (continuous proportion of residents in the HRR living below federal poverty thresholds), hospital type (university teaching hospital, community teaching hospital, and community non-teaching hospital) were included as HRR-level covariates. Covariates included in analysis were informed by previous studies [11, 12].

### Statistical analysis

Prevalence of GDM was reported for ethnic enclave residence and maternal characteristics, and by joint enclave-VOC exposure. Spearman rank correlations between each of the VOCs were calculated (Supplemental Tables 1 and 2).

Mothers in CSL were nested in HRRs for analysis. Hierarchical logistic regression models were used to estimate the odds ratio (OR) and 95% confidence intervals for the association between joint VOC/Enclave exposure and GDM, with robust standard errors to account for repeat births to the same mother (*n* = 731, 7.9% of births). Low VOC/Enclave exposure category served as reference group as we anticipated this was the lowest risk category. Separate models were run for each of the 14 VOCs for the preconception and first trimester exposure windows, using PROC GLIMMIX and PROC MIANALYZE (SAS 9.4) [65]. Benjamini-Hochberg false discovery rate adjustment procedure was used to account for multiple testing [66] (false discovery rate = 10%). Analyses were performed using PROC MULTTEST (SAS 9.4) [65].

### Sensitivity analyses

To further disentangle the potential effects of individual component measures (API population density, dissimilarity index or isolation index), we fit separate models to examine the association of ethnic enclaves, and each component part alone, with GDM. The ethnic enclave variable was dichotomous (yes/no), with 'no' serving as the reference category. The component variables were the tertile (low/medium/high) variables used to identify ethnic enclaves, with the 'low' category serving as the reference. Covariates included maternal age, marital status, health insurance, BMI, season of conception, parity, area-level poverty, hospital type, preconception benzene, and first trimester benzene.

Our primary models were single pollutant models but, we recognize that there is correlation between VOCs, and humans are typically exposed to a mixture of VOCs. We conducted a sensitivity analysis to determine if utilizing a measure of VOC mixtures modified results observed in the main analysis. We used Principal Component Analysis (PCA) to identify variables to include in a “multiple high VOCs” exposure category. PCA identified 7 VOCs that were jointly high: benzene, ethylbenzene, toluene, m-xylene, o-xylene, p-xylene, n-hexane. These 7 VOCs were jointly high for both preconception and first trimester exposure. If an individual was in the “high” group for all 7 of the VOCs, they were in the new “High Multiple VOC” group. We used this “high multiple VOC” group to identify new VOC-Enclave joint categories. Models with the same covariates as the primary models were run to estimate the association between High Multiple VOC/Enclave categories and GDM, with Not High in Multiple VOCs/Enclave areas serving as reference.

## Results

Of the 9069 pregnancies among API women in the CSL, there were 899 (9.9%) cases of GDM. Table 2 includes distribution of GDM by ethnic enclave residence, maternal characteristics, and area-level covariates. There were 1891 (20.8%) API women within ethnic enclaves, and 7178 (79.2%) API women in non-enclave areas. The prevalence of GDM was lower among women in ethnic enclaves (7.5%) compared to women in non-enclave areas (10.5%). GDM was more prevalent as BMI and age increased, as well as among multiparous women. GDM was more prevalent among women with private (10.6%) versus public (9.7%), self pay (9.3%) or other (6.5%) insurance coverage. GDM prevalence differed by season of conception, with warmer months having lower prevalence of GDM compared to colder months. Of note, GDM prevalence did not greatly differ by area-level poverty.

**Table 2:** Frequency (and percent) of GDM status by ethnic enclave residence and maternal characteristics among Asian/Pacific Islander women in the Consortium on Safe Labor among Asian/Pacific Islander women (*n* = 9069)

	Gestational Diabetes Mellitus		***p***-values^**a**^
	Yes (*n* = 899)	No (*n* = 8170)	
**Ethnic enclave**			
Yes (1891)	142 (7.5)	1749 (92.5)	*p < .01*
No (7178)	757 (10.5)	6421 (89.5)	
**Maternal Age**			
< 20 years (168)	4 (2.34)	164 (97.4)	*p < .01*
20–24 years (1289)	74 (5.7)	1215 (94.3)	
25–29 years (2797)	226 (8.1)	2571 (91.9)	
30–34 years (2958)	318 (10.8)	2640 (89.2)	
35+ years (1851)	277 (15.0)	1574 (85.0)	
Unknown/Missing [6]	0 (0.0)	6 (100.0)	
**Body Mass Index**			
≥ 30 (425)	75 (17.7)	350 (82.3)	*p < .01*
25–29.9 (744)	111 (14.9)	633 (85.1)	
18.5–24.9 (3466)	282 (8.1)	3184 (91.9)	
11.2–18.49 (621)	31 (5.0)	590 (95.0)	
Unknown (3813)	400 (10.5)	3413 (89.5)	
**Insurance Type**			
Private (6374)	677 (10.6)	5697 (89.4)	*p < .01*
Public (1280)	124 (9.7)	1156 (90.3)	
Self Pay (193)	18 (9.3)	175 (90.7)	
Other (1222)	80 (6.5)	1142 (93.5)	
**Marital Status**			
Married (7642)	800 (10.5)	6842 (89.5)	*p < .01*
Single (1241)	78 (6.3)	1163 (93.7)	
Divorced (186)	21 (11.3)	165 (88.7)	
**Parity**			
0 (4433)	395 (8.9)	4038 (91.1)	*p < .01*
≥ 1 (4636)	504 (10.9)	4132 (89.1)	
**Hospital Type**			
University Affiliated (3716)	329 (8.9)	3387 (91.1)	*p < .01*
Community: Teaching (4948)	541 (10.9)	4407 (89.1)	
Community: Non-teaching (405)	29 (7.2)	376 (92.8)	
**Season of Conception**			
March–May (2140)	203 (9.5)	1937 (90.5)	*p = .05*
June–August (2363)	208 (8.8)	2155 (91.2)	
September–November (2437)	250 (10.3)	2187 (89.7)	
December–February (2129)	238 (11.2)	1891 (88.8)	
**Area-Level Poverty**			
≥ 15.9% (3323)	348 (10.5)	2975 (89.5)	*p = .17*
< 15.9% (5746)	551 (9.6)	5195 (90.4)	

^a^*P*-values obtain using generalized estimating equations to account for women with > 1 pregnancy

Distribution of GDM by joint VOC/Enclave exposure categories is included in Table 3. For preconception VOC exposure, prevalence of GDM was lowest in Low VOC/Enclave areas for 7 of 14 VOCs, as anticipated, but was lowest in 6 of 14 High VOC/Enclave areas. For preconception exposure to sesquiterpene, Low VOC/Enclave areas and High VOC/Enclave areas, had the same GDM prevalence (7.5%). Prevalence of GDM was similar across categories of first trimester VOC exposure. For both preconception and first trimester exposures, non-enclave areas had higher GDM prevalence than enclave areas, regardless of VOC exposure levels.

**Table 3:** Frequency (and percent) of gestational diabetes mellitus status by joint preconception VOC-Enclave category among Asian/Pacific Islander women in the Consortium on Safe Labor (2002–2008)

			Preconception				First Trimester			
VOC (High = ≥ 75th percentile)		Enclave	n (***N*** = 9069)	GDM (***N*** = 899)	No GDM (***n*** = 8170)	***p*** value^**a**^	n (***N*** = 9069)	GDM (***N*** = 899)	No GDM (***n*** = 8170)	***p*** value^**a**^
Benzene	Low	Yes	242	13 (5.3)	229 (94.6)	*p < .01*	245	15 (6.1)	230 (93.9)	*p < .01*
		No	4115	415 (10.0)	3700 (90.0)		4179	420 (10.0)	3759 (90.0)	
	High	Yes	1649	129 (7.8)	1520 (92.2)		1646	127 (7.7)	1519 (92.3)	
		No	3063	342 (11.1)	2721 (88.9)		2999	337 (11.2)	2662 (88.8)	
Ethylbenzene	Low	Yes	245	15 (6.1)	230 (93.9)	*p < .01*	245	15 (6.1)	230 (93.9)	*p < .01*
		No	3916	406 (10.3)	3510 (89.7)		3943	412 (10.4)	3531 (89.6)	
	High	Yes	1646	127 (7.7)	1519 (92.3)		1646	127 (7.7)	1519 (92.3)	
		No	3262	351 (10.7)	2911 (89.3)		3235	345 (10.6)	2890 (89.4)	
MTB Ether	Low	Yes	738	63 (8.5)	675 (91.5)	*p < .01*	596	50 (8.4)	546 (91.6)	*p < .01*
		No	4578	437 (9.5)	4141 (90.5)		4436	419 (9.4)	4017 (90.6)	
	High	Yes	1153	79 (6.9)	1074 (93.1)		1295	92 (7.1)	1203 (92.9)	
		No	2600	320 (12.3)	2280 (87.7)		2742	338 (12.3)	2404 (87.7)	
N-hexane	Low	Yes	535	44 (8.22)	491 (91.8)	*p < .01*	410	32 (7.8)	378 (92.2)	*p < .01*
		No	3834	373 (9.7)	3461 (90.3)		3984	388 (9.7)	3596 (90.3)	
	High	Yes	1356	98 (7.2)	1258 (92.8)		1481	110 (7.4)	1371 (92.6)	
		No	3344	384 (11.5)	2960 (88.5)		3194	369 (11.5)	2825 (88.5)	
EMK	Low	Yes	739	58 (7.8)	681 (92.2)	*p < .01*	627	54 (8.6)	573 (91.4)	*p < .01*
		No	4827	490 (10.1)	4337 (89.9)		4615	460 (9.9)	4155 (90.1)	
	High	Yes	1152	84 (7.3)	1068 (92.7)		1264	88 (6.9)	1176 (93.1)	
		No	2351	267 (11.3)	2084 (88.7)		2563	297 (11.6)	2266 (88.4)	
m-xylene	Low	Yes	245	15 (6.1)	230 (93.9)	*p < .01*	245	15 (6.1)	230 (93.9)	*p < .01*
		No	3909	408 (10.4)	3501 (89.6)		3926	410 (10.4)	3516 (89.6)	
	High	Yes	1646	127 (7.7)	1519 (92.3)		1646	127 (7.7)	1519 (92.3)	
		No	3269	349 (10.7)	2920 (89.3)		3252	347 (10.7)	2905 (89.3)	
o-xylene	Low	Yes	304	18 (5.9)	286 (94.1)	*p < .01*	246	15 (6.1)	231 (93.9)	*p < .01*
		No	3897	405 (10.4)	3492 (89.6)		3936	410 (10.4)	3526 (89.6)	
	High	Yes	1587	124 (7.8)	1463 (92.2)		1645	127 (7.7)	1518 (92.3)	
		No	3281	352 (10.7)	2929 (89.3)		3242	347 (10.7)	2895 (89.3)	
p-xylene	Low	Yes	438	31 (7.1)	407 (92.9)	*p < .01*	338	24 (7.1)	314 (92.9)	*p < .01*
		No	3868	399 (10.3)	3469 (89.7)		3900	406 (10.4)	3494 (89.6)	
	High	Yes	1453	111 (7.6)	1342 (92.4)		1553	118 (7.6)	1435 (92.4)	
		No	3310	358 (10.8)	2952 (89.2)		3278	351 (10.7)	2927 (89.3)	
Propene	Low	Yes	827	67 (8.1)	760 (91.9)	*p < .01*	945	74 (7.8)	871 (92.2)	*p < .01*
		No	4728	449 (9.5)	4279 (90.5)		4471	420 (9.4)	4051 (90.6)	
	High	Yes	1064	75 (7.1)	989 (92.9)		946	68 (7.1)	878 (92.9)	
		No	2450	308 (12.6)	2142 (87.4)		2707	337 (12.5)	2370 (87.5)	
Sesquiterpene	Low	Yes	818	62 (7.5)	756 (92.5)	*p < .01*	741	62 (8.4)	679 (91.6)	*p < .01*
		No	3753	355 (9.5)	3398 (90.5)		3841	360 (9.4)	3481 (90.6)	
	High	Yes	1073	80 (7.5)	993 (92.5)		1150	80 (6.9)	1070 (93.1)	
		No	3425	402 (11.7)	3023 (88.2)		3337	397 (11.9)	2940 (88.1)	
Toluene	Low	Yes	334	19 (5.7)	315 (94.3)	*p < .01*	245	15 (6.1)	230 (93.9)	*p < .01*
		No	3891	407 (10.5)	3484 (89.5)		3934	411 (10.5)	3523 (89.5)	
	High	Yes	1557	123 (7.9)	1434 (92.1)		1646	127 (7.7)	1519 (92.3)	
		No	3287	350 (10.6)	2937 (89.4)		3244	346 (10.7)	2898 (89.3)	
Styrene	Low	Yes	736	64 (8.7)	672 (91.3)	*p < .01*	619	52 (8.4)	567 (91.6)	*p < .01*
		No	6268	657 (10.5)	5611 (89.5)		6266	661 (10.6)	5605 (89.4)	
	High	Yes	1155	78 (6.7)	1077 (93.3)		1272	90 (7.1)	1182 (92.9)	
		No	910	100 (11.0)	810 (89.0)		912	96 (10.5)	816 (89.5)	
1,3 butadiene	Low	Yes	732	64 (8.7)	668 (91.3)	*p < .01*	595	51 (8.6)	544 (91.4)	*p < .01*
		No	6197	642 (10.4)	5555 (89.6)		6354	673 (10.6)	5681 (89.4)	
	High	Yes	1159	78 (6.7)	1081 (92.3)		1296	91 (7.0)	1205 (93.0)	
		No	981	115 (11.7)	866 (88.3)		824	84 (10.1)	740 (89.9)	
Cyclohexane	Low	Yes	1022	66 (6.5)	956 (93.5)	*p < .01*	1016	74 (7.3)	942 (92.7)	*p < .01*
		No	3747	391 (10.4)	3356 (89.6)		3794	400 (10.5)	3394 (89.5)	
	High	Yes	869	76 (8.7)	793 (91.3)		875	68 (7.8)	807 (92.3)	
		No	3431	366 (10.7)	3065 (89.3)		3384	357 (10.6)	3027 (89.5)	

^a^*P*-values obtain using generalized estimating equations to account for women who had more than one pregnancy in the study

Hierarchical regression results for the association between VOC/Enclave joint exposure and GDM are reported in Table 4. Compared to Low VOC/Enclave areas, non-enclave areas were generally associated with higher risk of GDM, regardless of VOC exposure levels. For example, preconception benzene exposure was associated with elevated risk for High VOC/No Enclave (OR:3.45, 95%CI:1.77, 6.72) and for Low VOC/No Enclave (OR:2.85, 95%CI:1.57, 5.17), while the risk for High VOC/Enclave (OR:2.07, 95%:1.09, 3.94) was elevated but somewhat mitigated. There was a similar pattern for 12 of 14 VOC during preconception and 10 of 14 during the first trimester. For example, for propene exposure, risks were similar for both preconception High VOC/No Enclave (OR:1.99, 95%CI: 1.46, 2.72) and first trimester High VOC/No Enclave (OR:1.96, 95%CI: 1.44, 2.67).

**Table 4:** Joint associations between exposure to ambient volatile organic compounds, ethnic enclaves, and gestational diabetes mellitus among Asian/Pacific Islander women in the Consortium on Safe Labor (2002–2008)

			Preconception		First Trimester	
VOC (High = ≥ 75th percentile)		Enclave	n (***N*** = 9069)	Odds Ratio (95% CI)	n (***N*** = 9069)	Odds Ratio (95% CI)
Benzene	Low	Yes	242	Ref.	245	Ref.
		No	4115	2.85 (1.57, 5.17)^a^	4179	2.38 (1.36, 4.16)^a^
	High	Yes	1649	2.07 (1.09, 3.94)^a^	1646	1.65 (0.90, 3.03)
		No	3063	3.45 (1.77, 6.72)^a^	2999	2.70 (1.45, 5.03)^a^
Ethylbenzene	Low	Yes	245	Ref.	245	Ref.
		No	3916	2.43 (1.37, 4.32)^a^	3943	2.30 (1.29, 4.09)^a^
	High	Yes	1646	1.70 (0.90, 3.22)	1646	1.56 (0.82, 2.95)
		No	3262	2.76 (1.32, 5.75)^a^	3235	2.29 (1.10, 4.77)^a^
MTB Ether	Low	Yes	738	Ref.	596	Ref.
		No	4578	1.38 (1.02, 1.87)^a^	4436	1.40 (1.01, 1.95)
	High	Yes	1153	0.87 (0.58, 1.31)	1295	0.90 (0.60, 1.36)
		No	2600	1.84 (1.34, 2.52)^a^	2742	1.88 (1.33, 2.66)^a^
N-hexane	Low	Yes	535	Ref.	410	Ref.
		No	3834	1.56 (1.11, 2.20)^a^	3984	1.72 (1.16, 2.55)^a^
	High	Yes	1356	1.03 (0.68, 1.57)	1481	1.16 (0.74, 1.83)
		No	3344	2.09 (1.39, 3.15)^a^	3194	2.25 (1.43, 3.53)^a^
EMK	Low	Yes	739	Ref.	627	Ref.
		No	4827	1.62 (1.19, 2.20)^a^	4615	1.43 (1.04, 1.96)^a^
	High	Yes	1152	1.09 (0.72, 1.63)	1264	0.85 (0.57, 1.28)
		No	2351	1.84 (1.32, 2.58)^a^	2563	1.67 (1.18, 2.36)^a^
m-xylene	Low	Yes	245	Ref.	245	Ref.
		No	3909	2.33 (1.31, 4.15)^a^	3926	2.30 (1.29, 4.08)^a^
	High	Yes	1646	1.59 (0.84, 3.01)	1646	1.55 (0.82, 2.94)
		No	3269	2.40 (1.15, 5.00)^a^	3252	2.27 (1.08, 4.76)^a^
o-xylene	Low	Yes	304	Ref.	246	Ref.
		No	3897	2.37 (1.42, 3.98)^a^	3936	2.35 (1.32, 4.17)^a^
	High	Yes	1587	1.66 (0.94, 2.94)	1645	1.60 (0.85, 3.04)
		No	3281	2.52 (1.30, 4.87)^a^	3242	2.42 (1.15, 5.09)^a^
p-xylene	Low	Yes	438	Ref.	338	Ref.
		No	3868	1.87 (1.25, 2.78)^a^	3900	1.93 (1.23, 3.03)^a^
	High	Yes	1453	1.27 (0.80, 2.03)	1553	1.29 (0.77, 2.16)
		No	3310	2.02 (1.20, 3.40)^a^	3278	1.93 (1.06, 3.53)^a^
Propene	Low	Yes	827	Ref.	945	Ref.
		No	4728	1.44 (1.07, 1.93)^a^	4471	1.45 (1.09, 1.93)^a^
	High	Yes	1064	0.93 (0.64, 1.36)	946	0.94 (0.66, 1.34)
		No	2450	1.99 (1.46, 2.72)^a^	2707	1.96 (1.44, 2.67)^a^
Sesquiterpene	Low	Yes	818	Ref.	741	Ref.
		No	3753	1.55 (1.15, 2.10)^a^	3841	1.38 (1.02, 1.87)^a^
	High	Yes	1073	1.13 (0.77, 1.65)	1150	0.87 (0.59, 1.28)
		No	3425	2.23 (1.59, 3.14)^a^	3337	1.91 (1.36, 2.69)^a^
Toluene	Low	Yes	334	Ref.	245	Ref.
		No	3891	2.39 (1.45, 3.95)^a^	3934	2.31 (1.30, 4.11)^a^
	High	Yes	1557	1.68 (0.96, 2.91)	1646	1.57 (0.83, 2.97)
		No	3287	2.38 (1.25, 4.52)^a^	3244	2.31 (1.09, 4.89)^a^
Styrene	Low	Yes	736	Ref.	619	Ref.
		No	6268	1.41 (1.04, 1.93)^a^	6266	1.47 (1.05, 2.06)^a^
	High	Yes	1155	0.84 (0.56, 1.26)	1272	0.89 (0.59, 1.34)
		No	910	1.59 (1.13, 2.24)^a^	912	1.56 (1.08, 2.25)^a^
1,3 butadiene	Low	Yes	732	Ref.	595	Ref.
		No	6197	1.39 (1.02, 1.88)^a^	6354	1.48 (1.06, 2.06)^a^
	High	Yes	1159	0.83 (0.55, 1.25)	1296	0.86 (0.57, 1.29)
		No	981	1.67 (1.19, 2.33)^a^	824	1.46 (1.01, 2.13)^a^
Cyclohexane	Low	Yes	1022	Ref.	1016	Ref.
		No	3747	1.86 (1.37, 2.53)^a^	3794	1.70 (1.26, 2.28)^a^
	High	Yes	869	1.31 (0.92, 1.87)	875	1.08 (0.76, 1.54)
		No	3431	1.73 (1.15, 2.59)^a^	3384	1.46 (0.98, 2.18)

Analytic sample restricted to Asian/Pacific Islander women without diagnosed preconception diabetes. Hierarchical logistic regression, women nested within hospital referral region. Models adjusted for maternal age, preconception BMI, parity, insurance status, hospital, marital status, area-level poverty, season of birth. ^a^statistically significant after Benjamini-Hochberg procedure (false discovery rate = 10%)

Results of the enclave components sensitivity analysis are shown in Table 5. Residence in an ethnic enclave was associated with 49% lower odds of GDM (OR:0.51, 95%CI:0.37, 0.69) compared to residence in a non-enclave area. For the ethnic enclave components, high API population density was associated with a 38% lower odds of GDM (OR:0.62, 95%CI: 0.46, 0.83) compared to low API population density. Additionally, high levels of dissimilarity (OR: 0.81, 95% CI:0.64, 1.08) and isolation (OR: 0.83, 95% CI:0.64, 1.07) suggest a potential healthy effect compared to respective low levels.

**Table 5:** Sensitivity analysis: Association between ethnic enclaves and component measures and gestational diabetes mellitus

		Ethnic Enclave components			
	API Enclave OR (95% CI)		API Dissimilarity OR (95% CI)	API Isolation OR (95% CI)	API concentration OR (95% CI)
Non-Enclave	Ref.	Low	Ref.	Ref.	Ref.
Enclave	0.51 (0.37, 0.69)	Medium	0.83 (0.64, 1.08)	1.10 (0.88, 1.39)	0.79 (0.55, 1.12)
		High	0.81 (0.64, 1.08)	0.83 (0.64, 1.07)	0.62 (0.46, 0.83)

Models adjusted for maternal age, marital status, health insurance, BMI, season of conception, parity, area-level poverty, hospital type, preconception benzene, and first trimester benzene

The “high multiple VOC” sensitivity analysis results are consistent and presented in Supplemental Table 3. Compared to Not High in Multiple VOCs/Enclave areas, non-enclave areas were associated with higher risk of GDM, regardless of VOC exposure levels. Results were similar across exposure windows. For example, High in Multiple VOCs/Non-Enclave was associated with approximately 75% increased risk of GDM in preconception (OR: 1.74 95%CI: 1.12, 2.69) and first trimester (OR: 1.75 95%CI: 1.08, 2.86) exposure windows.

## Discussion

In this first investigation of the association between joint exposure to air pollution and residence in an ethnic enclave and GDM risk, we found evidence that residence within an ethnic enclave may mitigate negative consequences of environmental exposures. In line with evidence of an association between preconception and first trimester exposure to air pollution and increased risk of GDM [12, 28, 67–69] as well as evidence of lower risk of GDM among women residing within ethnic enclaves [11, 17, 18] we found evidence that residence in enclaves is associated with lower GDM risk, regardless of VOC level.

The observations suggest chronic exposure to residence outside of ethnic enclaves and VOCs are associated with increased GDM risk for API mothers, as risks appear consistent across preconception and first trimester exposure windows. Previously among women in the CSL, we have observed consistent increases in GDM risk across preconception and first trimester exposure windows for criteria air pollutants such as nitrogen oxides and sulfur dioxide [28], as well as VOCs [12]. Similar observations of chronic exposure to criteria air pollutants and GDM were observed among women in Denmark, Sweden, and Taiwan [67–69]. Given that air pollution and ethnic enclave exposures are likely chronic, the development of GDM is likely not due to an acute exposure in pregnancy.

As ethnic enclave residence appears to mitigate the negative consequences of VOC exposure, these observations suggest immunologic function may be an important factor. The normative immunologic response to air pollution, including during pregnancy [27], induces pro-inflammatory responses evidenced by heightened cytokine production and serum c-reactive protein levels [27, 32, 37–40]. Exposure to chronic stress leads to excessive release of stress hormones resulting in physiologic dysregulation, including impaired immune function, and consequent excessive inflammation [42, 43]. Evidence of immune function in regards to ethnic enclave residence is seen among Hispanic women, as those residing in ethnic enclaves have lower risk of allostatic load (dysfunction across multiple physiologic domains including impaired immune function), compared to those residing in non-enclave areas [20]. Impaired immunologic function may respond to air pollution exposure with excessive inflammation, resulting in excessive release of pro-inflammatory cytokines and damage to healthy cells, which in turn can lead to insulin resistance, a precursor to metabolic disease [41, 42]. Thus, the similar systemic inflammatory and oxidative stress responses between exposure to chronic stress and exposure to air pollution may explain the synergic effects between residence in non-enclave areas and exposure to high levels of VOCs.

Our findings are also in line with evidence suggesting that the deleterious effect of air pollution on health is stronger among those residing in more stressful contexts. For instance, the effect estimates for exposure to high levels of VOCs with poor cardiometabolic health are higher among adolescents residing in high-poverty areas compared to those residing in low-poverty areas [70]. Additionally, criteria air pollution exposure during the first year of life is associated with increased risk of childhood asthma, but only among children in high poverty areas [71]. It is noteworthy that the observed GDM risks were independent of individual-level proxies of health insurance and marital status, suggesting residence in an ethnic enclave may buffer the negative consequences of exposure to high levels of air pollution.

The results of the enclaves components sensitivity analysis suggest our measure of ethnic enclaves better depicts the unique social and geographic attributes of API ethnic enclaves than any of the individual components do alone (Table 5). While high levels of dissimilarity index, isolation index, and population density suggest a potential healthy effect, the healthy effect is greatest in those areas identified as API ethnic enclaves. The combination of dissimilarity index, isolation index, and population density allowed us to identify areas that were geographically distinct, and with a large enough population of API residents to be socially distinct. This definition of API ethnic enclaves could be refined by inclusion of more culturally-relevant data such as ancestry data, immigration data, and language data.

The results of the “high multiple VOC” analysis do not change the interpretation that ethnic enclaves are protective against the negative effects of air pollution. While PCA solves the potential VOC inter-correlation problem, it does not allow for examination of specific exposures that may be related to particular sources, or for confirmation from animal or occupational studies which might focus on individual compounds. Additionally, given the observed effects of the “high multiple VOC” approach greatly differs from several individual VOCs, this “high multiple VOC” approach may be masking especially harmful VOCs. Since examination of VOCs is novel in regards to GDM, we want to maintain the individual exposures. We believe this specificity can encourage occupational and animal studies to potentially confirm our findings.

Our observations highlight the importance of focusing on API communities in environmental health research. API communities are often aggregated in research and identified as ‘model minorities’ due to higher socioeconomic status compared to other non-white racial/ethnic groups in the U.S., suggesting API communities have favorable health outcomes compared to other racial/ethnic groups [6, 9]. Reliance on the ‘model minority’ label, in addition to API encompassing approximately 6% of the U.S. population, contributes to limited representation of API populations in national datasets, the homogenization of API ancestry by aggregation across distinct countries of origin, poor recognition of disparities among API populations, and a lack of environmental justice research targeting API communities [6–9]. By aggregating API ancestry groups, important differences in health status and environmental exposures may be masked, thus representing the same level of risk for poor health outcomes among a diverse group. The lack of relevant data excludes API communities from environmental health policy and health promotion planning when they may be an at-risk group [7, 9]. Given known health disparities, adverse environmental exposures, and the need for data disaggregation among API communities, public health surveillance and research should increase efforts to collect ancestry-specific and culturally-specific data to better address disparities impacting API communities.

In order to improve health outcomes among U.S. API populations, it could be beneficial for API communities to implement culturally-specific efforts to jointly improve social and environmental conditions. Previous attempts to improve environmental conditions have failed when a community’s cultural considerations have not been taken into account, resulting in worse environmental conditions and rapid displacement and gentrification [72–74]. API communities in California have been successful in community-led efforts to assemble multisector coalitions to implement environmentally friendly transportation and infrastructure improvements, affordable housing developments, and economic vitalization that reflect cultural values of API communities [72]. However, further research is warranted to better understand the population-health benefits of these community-led efforts.

Our findings are notable for several reasons. First, to the best of our knowledge, this is the initial investigation of joint exposure to air pollution and residence in an ethnic enclave among pregnant women. The observations that residence within an ethnic enclave mitigates air pollution suggest chronic exposure to low or high stress prior to pregnancy has important physiologic implications during pregnancy. Secondly, this study expands our understanding of complex socioenvironmental exposures among an understudied minority population. API communities are at greater risk for high air pollution exposure, and are typically concentrated within urban areas in the U.S. Lastly, this study benefits from a large amount of clinical data for a large sample of API women in the CSL. This allows for a robust examination of community-level risk factors for GDM, a condition that disproportionately affects U.S. API women.

These findings are best considered in the context of the study’s limitations. Our measure of ethnic enclaves has not been validated in studies outside of the CSL [11], as to the best of or our knowledge, no validated measure of ethnic enclaves exists. Given HRR is the sole geographic unit of analysis in the CSL, our measurement of ethnic enclaves was informed by previous studies in order to best capture social and geographic distinctions of ethnic enclaves. API women in the CSL are aggregated into a single category, not allowing us to examine API women by ancestry. Due to this, we used the aggregated API census data to measure ethnic enclaves. This limits our observations as API ancestry may be related to GDM risk [18], and air pollution exposure [6, 75], and effect of ethnic enclave residence may differ by API ancestry [17, 18]. However, previous analyses suggest the API population of metropolitan areas represented in the CSL is over 93% women of Asian ancestry with relatively few Pacific Islander women [12]. The CSL lacks maternal residential history, limiting our understanding of length of exposure to ethnic enclaves. However, most residential relocation during pregnancy occurs with a similar geographic area, and cross-sectional data allows for an approximate understanding of chronic exposures to community-level factors [76].

Immigration history for women within the CSL is not available, thus, examination of differences by immigration status is not possible. From our previous analysis of API ethnic enclaves in the CSL, the API population within metropolitan areas represented in the CSL is over 65% foreign born [11], suggesting potential acculturation to U.S. norms or a healthy migrant effect may affect our results. More detailed immigration data may allow for additional explorations of acculturation and healthy migrant in the context of ethnic enclaves.

Overall, we applied a conservative strategy for estimating VOC exposure, averaging over broader time and space dimensions to provide more stable estimates. Due to this approach, these observations are likely biased towards the null for several reasons. We averaged VOC exposure over the HRR, which reduces the impact of small point source exposure. We note that there is larger degree of uncertainty in CMAQ models with respect to population-level VOC exposures compared to exposure to ambient criteria air pollutants, such as PM and ozone. To account for this, we examined a dichotomous high/low VOC exposure variable as we did not assume VOCs were measured well enough to estimate linear relationships and there is no routine VOC monitoring data to fuse to the modified CMAQ data. We recognize a more robust continuous estimate may elucidate these relationships and better describe biologic mechanisms, as well as provide more information for regulatory decisions. We encourage other researchers with more robust VOC data, such as air pollution estimates from CMAQ-CB6 [77], to further analyze this question with better spatial resolution.

VOC exposure was averaged over HRRs in which the birth occurred and was not based on participant residence or specific location of ethnic enclaves. However, our enclave and exposure estimates are based on the areas covered by the HRR under the assumption that most women will live in the catchment area of their delivery hospital. Exposure misclassification may occur if mothers resided outside the HRR for all or part of their pregnancy. However, while 10–30% of pregnant women change residence during pregnancy, most move to an area of similar level of air pollution [78, 79]. Misclassification may also be a function of local mobility and activity patterns of pregnant women. While the CSL does not have local mobility or daily activity data, current evidence suggests pregnant women and a general population comparison group both spent approximately 15 h per day indoors at or near their home [80]. Additionally, during the first trimester of pregnancy, exposure estimates based on residential address are strongly correlated with exposure estimates accounting for daily activities (r = 0.98, *p* < 0.01) [81].

## Conclusions

In conclusion, we observed that API women residing in non-enclave areas have higher risk for GDM, regardless of VOC level. Residence in an ethnic enclave may mitigate the negative health effects of VOC exposure, potentially due to lower stress levels. Lower levels of stress among residents of ethnic enclaves may be related to greater access to culturally-relevant goods and services, and greater political representation [3, 4, 15, 24]. API communities should lead culturally-relevant efforts to promote health through improved social and environmental conditions. Additional research is warranted to better understand the effects of joint exposures to air pollution and ethnic enclave across diverse ancestry groups within the broader U.S. API population.

## Supplementary Information

**Additional file 1.**

## Data Availability

Consortium on Safe Labor data is publicly available at https://dash.nichd.nih.gov/. Geographic identifying information is not publicly available, please see http://grants.nih.gov/grants/policy/data_sharing/ for National Institutes of Health data sharing policy. Dartmouth Atlas of Healthcare data is available at https://www.darthmouthatlas.org/data

## Abbreviations

95% CI: 95% confidence intervals
ACS: American Community Survey
API: Asian/Pacific Islander
BMI: Body mass index
CSL: Consortium on Safe Labor
GDM: Gestational diabetes mellitus
HRR: Hospital referral region
OR: Odds ratio
U.S.: United States
VOC: Volatile organic compounds
ZCTA: Zip code tabulation area
