# Supplementary material for: Joint effects of ethnic enclave residence and ambient volatile organic compounds exposure on risk of gestational diabetes mellitus among Asian/Pacific Islander women in the United States
Source: Environ Health. 2021 May 8;20:56. doi: 10.1186/s12940-021-00738-7 (PMC8106843; doi:10.1186/s12940-021-00738-7)
Supplement: Supplementary file 1 — Additional file 1. [file 12940_2021_738_MOESM1_ESM.docx]

**Supplemental Table 1.** Spearman correlation coefficients for volatile organic compounds at 3 months prior to conception (n= 9,069) ^a^

|  | **Benzene** | **Ethylbenzene** | **MTB Ether** | **N-hexane** | **EMK** | **m-xylene** | **o-xylene** | **p-xylene** | **propene** | **Sesquiterpene** | **Toluene** | **Styrene** | **1,3 butadiene** | **Cyclohexane** |
| --- | --- | --- | --- | --- | --- | --- | --- | --- | --- | --- | --- | --- | --- | --- |
| **Benzene** | 1.00 | 0.97 | 0.59 | 0.96 | 0.85 | 0.95 | 0.90 | 0.95 | 0.53 | 0.71 | 0.87 | 0.10 | 0.12 | 0.80 |
| **Ethylbenzene** |  | 1.00 | 0.59 | 0.96 | 0.90 | 0.97 | 0.93 | 0.99 | 0.57 | 0.74 | 0.88 | 0.11 | 0.14 | 0.81 |
| **MTB Ether** |  |  | 1.00 | 0.66 | 0.82 | 0.44 | 0.29 | 0.62 | 0.93 | 0.52 | 0.18 | 0.74 | 0.76 | 0.10 |
| **N-hexane** |  |  |  | 1.00 | 0.89 | 0.91 | 0.87 | 0.96 | 0.60 | 0.78 | 0.82 | 0.18 | 0.20 | 0.77 |
| **EMK** |  |  |  |  | 1.00 | 0.80 | 0.71 | 0.91 | 0.82 | 0.68 | 0.63 | 0.47 | 0.50 | 0.52 |
| **m-xylene** |  |  |  |  |  | 1.00 | 0.98 | 0.95 | 0.42 | 0.71 | 0.95 | -0.07 | -0.05 | 0.89 |
| **o-xylene** |  |  |  |  |  |  | 1.00 | 0.91 | 0.26 | 0.67 | 0.99 | -0.21 | -0.19 | 0.95 |
| **p-xylene** |  |  |  |  |  |  |  | 1.00 | 0.60 | 0.76 | 0.86 | 0.16 | 0.19 | 0.78 |
| **propene** |  |  |  |  |  |  |  |  | 1.00 | 0.48 | 0.15 | 0.75 | 0.77 | 0.05 |
| **Sesquiterpene** |  |  |  |  |  |  |  |  |  | 1.00 | 0.65 | 0.27 | 0.25 | 0.59 |
| **Toluene** |  |  |  |  |  |  |  |  |  |  | 1.00 | -0.28 | -0.26 | 0.97 |
| **Styrene** |  |  |  |  |  |  |  |  |  |  |  | 1.00 | 0.99 | -0.37 |
| **1,3 butadiene** |  |  |  |  |  |  |  |  |  |  |  |  | 1.00 | -0.36 |
| **Cyclohexane** |  |  |  |  |  |  |  |  |  |  |  |  |  | 1.00 |

1. All correlations statistically significant p<.001

**Supplemental Table 2.** Spearman correlation coefficients for volatile organic compounds for first trimester of pregnancy (n= 9,069) ^a^

|  | **Benzene** | **Ethylbenzene** | **MTB Ether** | **N-hexane** | **EMK** | **m-xylene** | **o-xylene** | **p-xylene** | **propene** | **Sesquiterpene** | **Toluene** | **Styrene** | **1,3 butadiene** | **Cyclohexane** |
| --- | --- | --- | --- | --- | --- | --- | --- | --- | --- | --- | --- | --- | --- | --- |
| **Benzene** | 1.00 | 0.97 | 0.65 | 0.97 | 0.87 | 0.95 | 0.92 | 0.96 | 0.58 | 0.72 | 0.88 | 0.07 | 0.10 | 0.81 |
| **Ethylbenzene** |  | 1.00 | 0.65 | 0.96 | 0.91 | 0.98 | 0.94 | 0.99 | 0.61 | 0.73 | 0.89 | 0.08 | 0.10 | 0.82 |
| **MTB Ether** |  |  | 1.00 | 0.72 | 0.84 | 0.52 | 0.39 | 0.67 | 0.92 | 0.58 | 0.28 | 0.65 | 0.66 | 0.19 |
| **N-hexane** |  |  |  | 1.00 | 0.90 | 0.92 | 0.88 | 0.96 | 0.64 | 0.77 | 0.84 | 0.14 | 0.16 | 0.78 |
| **EMK** |  |  |  |  | 1.00 | 0.83 | 0.75 | 0.92 | 0.83 | 0.68 | 0.66 | 0.40 | 0.42 | 0.56 |
| **m-xylene** |  |  |  |  |  | 1.00 | 0.98 | 0.96 | 0.48 | 0.69 | 0.95 | -0.09 | -0.07 | 0.90 |
| **o-xylene** |  |  |  |  |  |  | 1.00 | 0.92 | 0.34 | 0.66 | 0.99 | -0.21 | -0.19 | 0.94 |
| **p-xylene** |  |  |  |  |  |  |  | 1.00 | 0.63 | 0.75 | 0.87 | 0.12 | 0.15 | 0.79 |
| **propene** |  |  |  |  |  |  |  |  | 1.00 | 0.49 | 0.23 | 0.66 | 0.68 | 0.13 |
| **Sesquiterpene** |  |  |  |  |  |  |  |  |  | 1.00 | 0.64 | 0.26 | 0.24 | 0.57 |
| **Toluene** |  |  |  |  |  |  |  |  |  |  |  | -0.27 | -0.25 | 0.96 |
| **Styrene** |  |  |  |  |  |  |  |  |  |  |  | 1.00 | 0.99 | -0.36 |
| **1,3 butadiene** |  |  |  |  |  |  |  |  |  |  |  |  | 1.00 | -0.35 |
| **Cyclohexane** |  |  |  |  |  |  |  |  |  |  |  |  |  | 1.00 |

1. All correlations statistically significant p<.001

**Supplemental Table 3. Joint associations between exposure to multiple high VOCs, ethnic enclaves and gestational diabetes among Asian/Pacific Islander women in the Consortium on Safe Labor (2002-2008)**

|  | | | **Preconception** | | | **First Trimester** | | |  |
| --- | --- | --- | --- | --- | --- | --- | --- | --- | --- |
|  | | **Enclave** | **n**  **(N = 9069)** | **Odds Ratio**  **(95% CI)** |  | | **n**  **(N = 9069)** | **Odds Ratio**  **(95% CI)** | |
| High in Multiple VOCs | No | Yes | 625 | Ref |  | | 413 | Ref. | |
|  |  | No | 4337 | 1.69 (1.21, 2.34) |  | | 4473 | 1.72 (1.16, 2.56) | |
|  | Yes | Yes | 1266 | 1.11 (0.73, 1.66) |  | | 1478 | 1.12 (0.71 (1.76) | |
|  |  | No | 2841 | 1.74 (1.12, 2.69) |  | | 2705 | 1.76 (1.08, 2.86) | |
